# Impact of Data Preprocessing on Integrative Matrix Factorization of Single Cell Data

**DOI:** 10.3389/fonc.2020.00973

**Published:** 2020-06-23

**Authors:** Lauren L. Hsu, Aedin C. Culhane

**Affiliations:** ^1^Department of Biostatistics, Harvard T.H. Chan School of Public Health, Boston, MA, United States; ^2^Division of Biostatistics and Computational Biology, Department of Data Sciences, Dana-Farber Cancer Institute, Boston, MA, United States

**Keywords:** data integration, matrix factorization, single cell, scRNA-seq, normalization, standardization, data preprocessing

## Abstract

Integrative, single-cell analyses may provide unprecedented insights into cellular and spatial diversity of the tumor microenvironment. The sparsity, noise, and high dimensionality of these data present unique challenges. Whilst approaches for integrating single-cell data are emerging and are far from being standardized, most data integration, cell clustering, cell trajectory, and analysis pipelines employ a dimension reduction step, frequently principal component analysis (PCA), a matrix factorization method that is relatively fast, and can easily scale to large datasets when used with sparse-matrix representations. In this review, we provide a guide to PCA and related methods. We describe the relationship between PCA and singular value decomposition, the difference between PCA of a correlation and covariance matrix, the impact of scaling, log-transforming, and standardization, and how to recognize a horseshoe or arch effect in a PCA. We describe canonical correlation analysis (CCA), a popular matrix factorization approach for the integration of single-cell data from different platforms or studies. We discuss alternatives to CCA and why additional preprocessing or weighting datasets within the joint decomposition should be considered.

## Introduction

Single-cell (sc) molecular profiling provides unprecedented resolution and incredible potential to discover the heterogeneity of cell types and states and intercellular communication that drives complex cellular dynamics, homeostasis, response to environment, and disease. We will focus this review on the challenges and considerations when applying matrix factorization approaches to integration of sc RNA sequencing data (scRNA-seq). Matrix factorization methods, including principal component analysis (PCA), are central to scRNA-seq data analysis pipelines, but are often treated as “black boxes” within computational pipelines, with little consideration of what steps are included. We will “open the box” to illustrate the exact scaling and transformations that are performed on data in a PCA, and how different preprocessing steps impact data and cross-platform batch integration. These tips and considerations will also apply other single cell omics data, as well as to multi-modal integration of different omics data.

### Challenging Properties of Single Cell Data

Single-cell data present a set of unique challenges for data analysis and integration (1–3). In contrast to traditional bulk RNA-seq which provides the average expression of RNA molecules across tens of thousands or millions of cells, scRNA-seq measures RNA in each cell.

The goal of scRNA-seq is frequently to define differential gene expression within specific cell types that characterize a phenotype, so cell type identification is a critical early step. In a tissue or biological sample, the population of cells is heterogeneous, containing many cell types including unidentified, new cell types, and cell states. Annotation of cell types in biological samples is challenging, as methods are still emerging and are limited by a lack of gold standard benchmarking data. To classify cell types and states, unsupervised clustering analysis is often used to partition cells into clusters, however, the biologically expected cell-to-cell variation within cell states is poorly understood, and cell clusters may be associated with systematic, batch, technical, or methodological artifacts (1). Toward the goal of creating a comprehensive cell type and state reference, the Human Cell Atlas will catalog the diversity of cell types in the human body (4) and anticipates discovering distinct tissue-specific, disease-specific, age-specific, gender-specific cell phenotypes, and will identify many new cell types and states that are yet to be defined.

Most, or at least half, of the transcriptome, is detected in a typical bulk RNAseq study. In contrast, scRNA-seq studies frequently measure <5,000 genes in a single cell (1). Most genes are not measured and these zero counts may represent zero gene expression or false negative dropout, that is, when a gene was expressed but was not detected due to technological limitations (3, 5) such as limited sequencing depth or stochastic variation. Gene expression may also be missed due to biological variance; single point-in-time measurements cannot capture dynamic processes, such as RNA transcriptional bursts. Emerging evidence suggests transcription occurs in bursts or pulses that depend on core promoter and enhancers (6) and a three-state model may be required to capture its biological complexity (7). These issues of scRNA-seq analysis underscore the importance of appropriate quality control, preprocessing, and normalization (1, 8).

### Preprocessing of sc Sequencing Data

Several library preparation and read mapping approaches including genome or transcriptome mapping and pseudo-alignment can be used to generate a “raw” or unique molecular identifier (UMI) count matrix from sequencing reads (9), but in a comparison of over 3,000 preprocessing and analysis pipelines, Vieth et al. found normalization of the count matrix had greatest impact on downstream analysis (9). Standard “normalization” pipelines include scaling using sample-specific size factors, log transformation to reduce skewness, and feature filtering before PCA. The selection of a particular normalization routine will itself embed assumptions about the underlying distribution of the data. Inappropriate preprocessing may introduce artifacts that impact the ability to perform further preprocessing (e.g., alignment and integration of batches of sc data both within and between studies) and downstream biological analyses [e.g., cell type identification, classification, and differential gene expression (1, 8, 9)].

**Figure 1:**
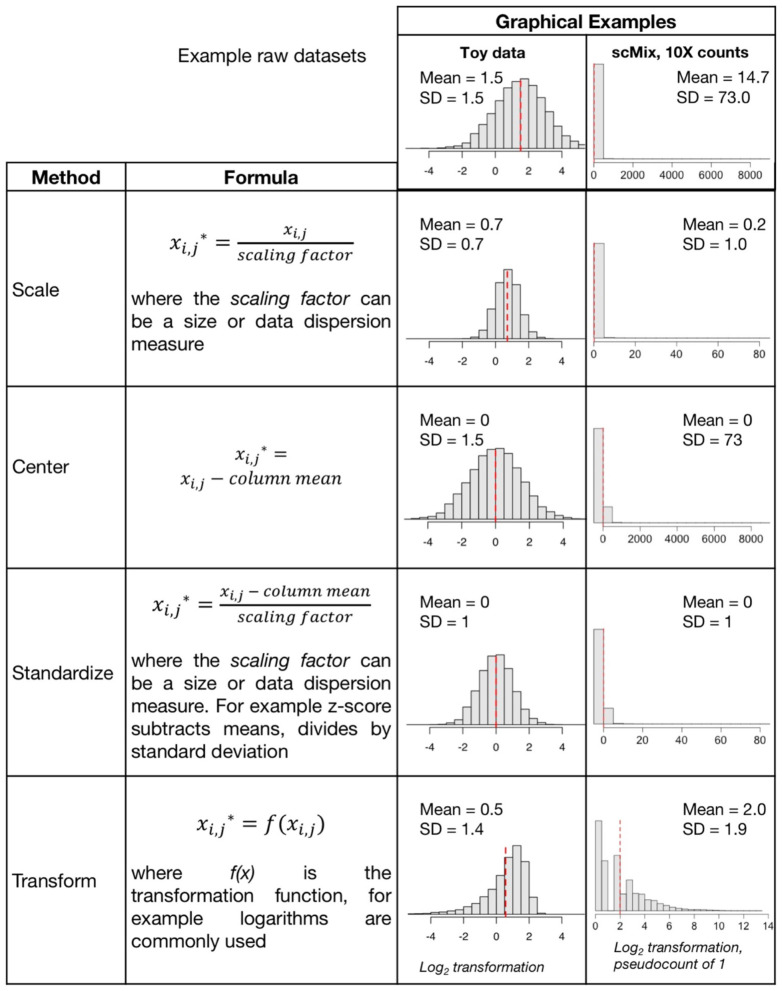
Common data preprocessing steps include scaling, centering, standardization, and transformation. Graphical examples of these preprocessing routines are applied to two datasets (1) “toy data” with a mean and standard deviation (SD) of 1.5 generated for purposes of illustration, and (2) the 10X raw counts matrix in the scMix benchmarking dataset used in Figure 2 (10).

Depending upon the analysis method selected, objective defined, and the dataset itself, different approaches to preprocessing may be appropriate; various data scaling, centering, standardization, and transformation (Figure 1) approaches can be applied. Frequently these terms are used interchangeably even though they represent different data manipulations (11, 12). Often the goal of preprocessing steps is to generate data that meet the linearity, homoscedasticity (that the points have the same scatter, i.e., there is no relationship between mean and variance), and normality assumptions that are required for most parametric statistical methods, including linear regression. A recent review of metabolomics data includes an extensive review of scaling and transformation approaches on sparse data (13).

- *Scaling* adjusts the range of the data, by dividing by a value. There are two broad subclasses of scaling factors: size measures (e.g., mean or library size) and data dispersion measures (e.g., standard deviation). Unit or unit variance scaling uses the standard deviation as the scaling factor, such that points have a standard deviation of one and therefore the data are analyzed on the basis of correlations instead of covariances. If data are scaled by dividing by the standard deviation, then the correlation is equal to the covariance of those two variables, since the Pearson correlation coefficient of two variables is equal to dividing the covariance of these variables by the product of their standard deviations. Scaling by size measures is important when integrating multiple datasets in cases where the range of values and means of the data differ substantially.
- *Centering* is subtracting the mean of a set of points from each data point so that the new mean is 0. The scale does not change, one unit is still one unit. In Figure 1, we see centering produces data with a mean at zero, but the standard deviation is unchanged
- *Standardization* includes *centering* and scaling. A *Z-score standardization* is subtracting the mean and dividing by the standard deviation of all points. A one-unit difference after this adjustment now indicates a one-standard deviation difference. Note whilst it changes the range of the data it may not affect the distribution, and may require an additional transformation
- *Transformations*, including log transformations (log_2_ or log_10_) or log with pseudocount (e.g., log +1), are commonly applied to sc data that increase proportionally (% or fold change) rather than linearly (8). A log transform or power transform may make skewed data look more symmetric or Gaussian (normally distributed in a bell-curve shape) and correct for heteroscedasticity (unequal scatter of points, where variance differs with mean). Recent studies reported that log_2_+1 transformation may distort data, introducing false variability in dimension reduction and impacting downstream analysis (8, 14, 15). Given that heteroscedasticity in omics data is both multiplicative and additive, generalized log variance-stabilizing transformations such as arcsinh (asinh) of scRNA-seq data (16, 17) and CyToF proteomic data (18, 19) are recommended. Rank-based inverse normal transformation has also been used to rescale scRNAseq gene expression (20).
- *Normalization* transforms the data points so that their distribution resembles a normal, also called Gaussian, distribution. In a normal distribution (i.e., the classic bell curve) points are distributed symmetrically around the mean, most observations are close to the mean, and the median and mean are the same. Depending upon the distribution of the original dataset, this may be achieved by a log transformation, or may require more extensive preprocessing. Two recent articles have proposed analysis of Pearson residuals rather than log normalized counts (8, 14). In bioinformatics and computational fields, this term may also refer to size and/or range scaling transformation which may not produce a normal distribution (21).

Feature selection, for instance restricting analysis to over-dispersed genes which are expected to capture a disproportionate amount of the variance in the data, is included in many analysis pipelines to reduce the computation time (16, 22). Furthermore, selecting genes with high biological variability, to exclude many genes with low biological signal and high numbers of zeros, may increase the signal to noise ratio in dimension reduction.

### Dimension Reduction

Data dimension reduction is indispensable in single cell data analyses because it facilitates exploratory data analysis and visualization, and is an essential step in many downstream analysis including cell clustering (23, 24), cell-type identification, cell trajectory, lineage reconstruction, and trajectory inference (25–27). It is also a critical first step in many algorithms that align and integrate sc datasets (11, 22, 28).

Dimension reduction transforms the data to a new coordinate system (i.e., a low-dimensional shared latent space) such that the greatest variance can be identified and distinguished from background noise, or less informative variance. The output is a set of embeddings for each data point which encode their location in the low-dimensional shared latent space. It is frequently achieved using matrix factorization, a class of unsupervised techniques that provide a set of principled approaches to parsimoniously reveal the low-dimensional structure while preserving as much information as possible from the original data.

Principal component analysis (PCA) is arguably the oldest, fastest, and the most commonly used matrix factorization approach (29). PCA is a deterministic algorithm that seeks linear combinations of the variables that explain the variance in the data and ranks these such that the first component explains most of the variance or “strongest” pattern in the data. PCA uses a Gaussian likelihood and is best applied to data that are approximately normally distributed. Whilst it is not recommended to be applied to highly skewed data (Figure 1), nonetheless, in a recent systematic analysis of 18 linear and non-linear dimension reduction approaches, PCA and other classical linear methods performed surprisingly well in both clustering and lineage inference analysis when assessed on 30 scRNA-seq datasets (30). Linear (straight-line) analysis methods including PCA, independent component analysis (ICA), factor analysis (FA) ranked best in clustering. PCA, FA, non-negative matrix factorization [NMF, (31, 32)], and uniform manifold approximation and projection [UMAP, (33)] ranked top in lineage inference analysis (30). We compare ICA and NMF matrix factorization in a recent review (31).

Dimension reduction methods optimized for count data that apply a better-fitting likelihood model (e.g., Poisson or negative binomial) are promising for addressing the skewed distribution of sc count data (8, 14). However, glmPCA (8), Poisson factorization (34–36), and probabilistic count matrix factorization [pCMF, (37)], as well as methods designed to model zero-inflated sparse data, including ZIFA and ZINB-WaVE (38, 39) did not outperform PCA across the full range of analyses and evaluations performed in the study Sun et al. (30). While there are particular settings where these methods may be most appropriate, they are not necessarily appropriate as “general-purpose” approaches. The high computational cost and long run time make many of these models difficult to integrate into multi-step bioinformatics pipelines.

Non-linear dimension reduction methods can identify variance in subsets of features by fitting local linear maps on subsets of points. Non-linear methods applied to sc data include diffusion maps (40), locally linear embedding, isoMap, kernel adaptations of linear methods, uniform manifold approximation and projection (UMAP) (41), and t-distributed stochastic neighbor embedding [tSNE, (42)]. However, similar to the methods that apply non-Gaussian likelihoods, non-linear dimension reduction methods are often computationally expensive and since they are not deterministic may produce different embeddings when re-applied to the same dataset. To improve computational tractability, PCA is frequently used as a preprocessing step prior to non-linear dimensionality reduction approaches including t-distributed stochastic neighbor embedding [tSNE, (43)] and UMAP (33). Although not required to run UMAP, in practice, it can be applied to accelerate computation time by significantly reducing dimensionality and noise while preserving underlying latent structure.

**Figure 2:**
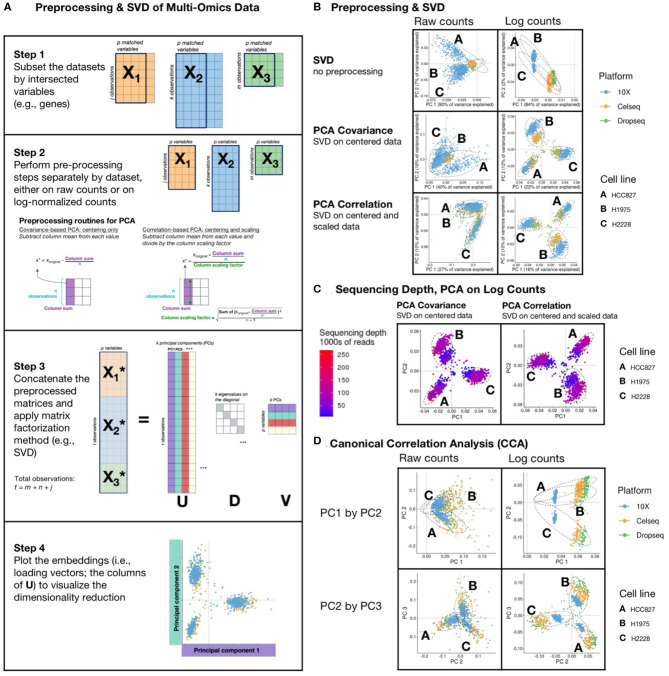
Matrix Factorization of sc data: **(A)** schematic diagram of a PCA or CCA workflow, includes: (1) filtering of datasets to intersecting genes; (2) scaling, transformation, and normalization of individual and joint count matrices; (3) concatenating matrices and applying a matrix factorization, usually singular value decomposition (SVD); and (4) visualizing results. SVD is a matrix operation that finds for a given input matrix the left singular vectors (U), the right singular vectors (V), and the singular values (D), such that the product of U and V with their respective transpose matrices is the identity matrix. Each singular vector is orthogonal to the others, and they are ordered such that the first component explains the greatest variance, and each subsequent component explains less than the preceding. **(B)** The first two principal components of SVD performed on counts and log-transformed counts of the scMix benchmarking data (10), comprising 3 cell lines (HCC827, H1975, and H2228), that were unprocessed, centered, and centered and scaled, to reflect SVD, covariance-based and correlation-based PCA, respectively. Results from covariance-based and correlation-based PCA applied to log-transformed data are similarly effective, showing moderate data integration and separation by cell type but an arch effect is visible on PC1 and PC2 in SVD of the raw counts. **(C)** Covariance-based and correlation-based PCA of log-transformed data, colored by sequencing depth, show that unadjusted differences in sequencing depth limit integration, forming a gradient across each cluster. **(D)** The first three principal components from Canonical Correlation Analysis (CCA) of scMix data. In both raw counts and log-transformed data, PC1 provides poor separation by cell type and batch integration. The plot of PC2 by PC3 from CCA on log-transformed data show reasonable clustering by cell line, though exhibit poor batch integration; in contrast, PC2 by PC3 plot from CCA on raw data shows better batch integration and poorer separation by cell type.

In this review, we focus on PCA because of its popularity, performance, and widespread use. PCA is a central step in many sc analysis algorithms and pipelines. When used with sparse-matrix representations, it can easily scale to large datasets. Excellent general tips for dimension reduction have been described (44), so we will focus on considerations and limitations when applying dimension reduction to sc data, including a step-by-step explanation of how PCA works, especially when applied to integrative sc analysis (Figure 2A).

#### The Impact of Data Preprocessing on Dimension Reduction

There are two types of PCA, which differ in data centering and scaling prior to matrix decomposition. PCA of a covariance matrix or a correlation matrix is achieved by applying matrix factorization to a centered but unscaled matrix, or a centered and scaled matrix, respectively (Figure 2A, Step 2). The latter is the most popular form of PCA. Linear regression using non-linear iterative partial least-squares (NIPALS), eigen analysis, or singular value decomposition (SVD) are a few of the many ways to factorize or decompose a matrix. SVD is a basic matrix operation, and fast approximations of SVD, including IRLBA, are commonly applied to sc data [extensively reviewed by (45)]. SVD factors an input matrix into three matrices U, D, and V, as illustrated schematically in Figure 2A (46) (R code to perform PCA via both eigen analysis and SVD are provided in Supplementary Methods). The maximum number of principal components or rank of the analysis is the number of rows or columns of the matrix (whichever is lower, n-1, or p-1), though typically 30 or fewer components are examined in most scRNA-seq pipelines (22). Selection of the correct number of components is non-trivial and most commonly achieved by heuristic approaches. To understand the distribution of variance explained by each component, scree-plots can also be helpful visual tool (47, 48) and permutations based approaches are recommended (49, 50).

Figure 2B displays SVD of raw count or log_2_ transformed count matrices that were (1) unprocessed data (top row); (2) centered by subtracting column means (middle row); and (3) scaled and centered to reproduce SVD. (2) and (3) show PCA of a covariance matrix (princomp in R), and PCA of a correlation matrix (prcomp in R), respectively (Figure 2B). These are applied to a small, well-described benchmarking dataset (10), comprising scRNA-seq measurements of a three cell line mixture on three technological platforms (10X, Dropseq, and CELseq2). Both forms of PCA had greater success in finding structure in the data as compared to SVD alone. However, clusters of cell lines could only be distinguished in data that were log transformed. Moderate cross platform integration was observed in data that were centered, or centered and scaled (equivalent of PCA of a covariance or correlation matrix, respectively). Nonetheless, as illustrated in Figure 2C, we observe that systematic differences in sequencing depth between the three platforms still creates a gradient across each cluster, preventing full integration. Whilst this analysis was performed on all variables (genes), we and others have found that excluding genes with low variability and high numbers of zeros prior to dimensionality reduction may increase the signal to noise ratio (12, 48, 51).

#### The Horseshoe or Arch Effect

PCA is optimized for continuous, normally distributed data and is suboptimal when applied to sparse data with many zero counts. The arch or horseshoe is a common pitfall and has been described in detail in the literature (44, 52, 53). This distortion results from the presence of a gradient or sequential latent ordering in the data [Tutorial by (54)]. In the top row of Figure 2B all of the cell lines on the first component (PC1) are on the same side of the origin, forming a classical horseshoe pattern, characterized by a distinctive “arched” shape, with points mostly on one side of the origin and folding back on itself in one of the dimensions. This indicates that additional data preprocessing is required; cell lines cannot be distinguished, and the data are not integrated across batches. In the top right plot of Figure 2B which shows SVD on unprocessed log counts, the first 2 PCs appear correlated, but are by definition orthogonal—their dot product is 0. Orthogonal vectors are uncorrelated only when at least one of them has mean 0. In contrast, when data are centered (e.g., middle and bottom row of Figure 2B), these artifacts are gone. It is vital that such arch effects are identified, especially when PCA forms part of a computational workflow that extracts the first *n* principal components without inspection. As seen in Figure 2, preprocessing and data normalization can remove arch artifacts and we refer the reader to excellent recent reviews on the subject (44, 52–54).

Examining PC plots can illuminate issues beyond the arch effect, in this case for instance, showing that the 10X data are located further from the origin on PC1 and PC2 as a result of difference in sequencing depth between platforms (Figures 2B,C). This can be corrected for by scaling the size factors by dataset to account for these systematic differences prior to log-normalization (55).

### Integrating Two or More Datasets With K-table Matrix Factorization

Matrix factorization approaches have been highly effective and widely applied to removing batch effects in bulk omics data (56, 57). Whilst dimensionality reduction methods like PCA can discover batch effects (1, 11, 28), and could also be applied to remove within or even between batch effects in sc data, it is more common to explicitly define the blocks, groups, or datasets to be integrated and apply matrix factorization that is designed to extract correlated structure between groups. Emerging sc data integration and cross-study batch correction methods frequently use PCA or joint matrix decompositions as a first step.

Matrix factorization approaches that integrate multiple groups or matrices with matched rows or columns, often called K-table, multi-block component analysis or tensor decompositions (46), have been applied to both bulk and scRNA-seq data integration (46). The simplest K-table approach is possibly Procrustean analysis (58, 59). Procrustes was a figure from Greek mythology who was famous for cutting limbs or stretching unknowing passers-by such that they fit into his bed, and similarly, Procrustean analysis involves rotation or reduction of a component from one PCA to best fit a second PCA. Several other matrix factorization approaches for K-table exist (46).

Arguably the most popular K-table approach applied to omics data is canonical correlation analysis [CCA, (60, 61)], which maximizes the correlation between components, or canonical variables of each dataset, and has been widely applied to integration of bulk omics data [reviewed by (46, 62)]. Classical CCA requires more observations than features, and therefore sparse implementations that include feature selection are used in the analysis of bulk omics data (63, 64). CCA and adaptations of CCA have been applied to integrate scRNA-seq including the cross-study integration of stimulated and resting human peripheral blood mononuclear cells (PBMCs); cross-platform integration of mouse hematopoietic progenitors scRNA-seq data; and heterogeneous case-control cell populations after drug exposure (16, 22). Seurat 3 uses CCA with anchors to align datasets that are extracted using mutual nearest neighbors on the CCA subspace (65). Harmony uses PCA as a first step (66). PCA or CCA is the first step in scAlign, a neural-network based method for pairwise or data to references, alignment of single cell data (67) which was reported to outperform other single cell alignment methods (CCA in Seurat, scVI, MNN scanorama, scmap, MINT, and scMerge). Non-linear matrix factorization approaches for integration of datasets include joint NMF [LIGER, (68)] but in a recent comparative study this was reported to be computationally slow and may overlay samples of little biological resemblance compared to the other methods (69). A benchmark comparison of 14 methods for integration of scRNA-seq datasets, on datasets from different technologies with identical cell types, non-identical cell types, multiple batches, big data, and simulated data revealed that harmony, LIGER, and Seurat 3 CCA are most performant (65).

Other matrix decomposition approaches, including multiple co-inertia analysis (48, 70), multiple factor analysis (71, 72), and consensus PCA (73–75), maximize a covariance or squared covariance criterion and are not limited by a requirement for more observations than features. These have been applied to bulk omics data and clustering, for example Meng et al., applied Westerhuis's modified implementation of consensus PCA to integrate methylation, proteomic and genomics data, reporting it was performant and faster that iCluster/iCluster+ (75). Dimension reduction methods for both single and K-table analysis, including a summary of the mathematical formulae and overview of available software packages for each mode of analysis, have been recently reviewed (46). Of note, there is also a recently described generalized framework to easily modulate between covariance and correlation-optimization in integrative matrix factorization (62, 76).

#### Horseshoes in CCA

Similar to PCA, a problematic arch effect is seen on PC1 and PC2 (Figure 2D) when CCA is applied to align and integrate raw counts or log counts of scRNA-seq measurements of three cell lines that were obtained on three technological platforms: 10X, Dropseq, and CELseq2 (10). The raw data had more platform overlap, and the log-transformed had less overlap in cell types in PC2 and PC3 (Figure 2D). These data demonstrate that, if CCA is used as a first step in a pipeline, it should include a check for the presence of such artifacts. For example, upon examining Figure 2D, one could exclude PC1, since CCA integrates the data across platforms in PC2 and PC3.

#### Scaling of Datasets in CCA

Simultaneous integration of multiple matrices is more complex than integrative analysis of a single dataset because each dataset may have different numbers of observations (cells), internal structure, and variance. In this CCA (Figure 2D) vignette the 10X dataset exhibited less correlated structure with the Dropseq and CELseq2 datasets, which had lower sequencing depth (Figure 2C). Therefore, in K-table matrix decomposition two levels of preprocessing are recommended. First, each individual dataset is normalized, centered, and scaled. Secondly, datasets are scaled by cross-dataset size factors (55), weighted to inflate or deflate the contribution of individual datasets, such as scaling by the square root of their total inertia, the percent variance on the first principal component, sample size, or another measure of data quality or expected contribution [reviewed by (46)].

### Key Takeaways

When applying matrix factorization methods including PCA, it is recommended to consider the impact of scaling, log-transforming, standardization, and normalization. Common data challenges, and tips to address them, include:

1. *Preprocessing of data*. Consider each step in the pipeline and how it transforms the data. If necessary, consider preprocessing the data yourself. *Visualize data* after intermediate steps to ensure data are processed as expected, and to diagnose any issues that may arise.
2. *Heteroscedasticity*. Whilst widely used, log_2_ transformation of expression values combined with pseudocounts may not be appropriate, consider using a variance-stabilizing transformation.
3. *Arch effect in PCA*. Examine PCs if weights are not centered around the origin with negative and positive scores, to check if there is an arch artifact. This can be mitigated by scaling and/or normalization.
4. *Systematic differences in sequencing depth*. When working with data from multiple batches, we found that the *multiBatchNorm* function from the *batchelor* R/Bioconductor package corrected for the differences in sequencing depth.
5. *Uncertainty around ground truth*. Test methods using a well-characterized benchmarking dataset, if possible. The *CellBench* R/Bioconductor package provides access to several datasets, including the scmix dataset used in Figure 2 (77).

## Summary

Single cell omics data are expanding our understanding of tumor heterogeneity, the tumor microenvironment, and tumor immunology. Algorithms for cell clustering, cell type identification, and cell trajectory analysis rely on dimension reduction to achieve computationally tractable solutions. The sparsity, noise, and high dimensionality of these data present unique challenges and underscore the importance of dimension reduction in sc analysis. PCA is widely used and popular for its speed, scalability, and performance, though it may not be the most optimal method for sc data. Matrix factorization approaches optimized for count matrices or distances matrices have been described [reviewed by (38)], and it is likely that more performant data preprocessing, scaling, and transformation approaches will continue to be developed. These methods will improve the performance of dimension reduction approaches in sc data integration and analysis.

## Resources

We include below a short list of single cell analysis resources, vignettes, and reference materials

https://osca.bioconductor.org/
https://github.com/seandavi/awesome-single-cell
https://satijalab.org/seurat/
https://hemberg-lab.github.io/scRNA.seq.course/
https://github.com/SingleCellTranscriptomics

## Supplemental Material

R Code to reproduce these figures which describes different implementation of SVD and PCA is publicly available at https://github.com/aedin/Frontiers_Supplement/. It includes a code to generate PCA, computed by SVD, eigenanalysis and PCA using R packages princomp, prcomp, ade4, FactoMineR. In each case, the relationship between these methods is described.

## Author Contributions

LH and AC wrote the paper. LH wrote the code and performed analysis. AC wrote the online supplemental PCA vignette code.

## Conflict of Interest

The authors declare that the research was conducted in the absence of any commercial or financial relationships that could be construed as a potential conflict of interest.
